# Incidence, Predictors, and Outcomes of Major Transcatheter Aortic Valve Replacement (TAVR) Complications and Failure-to-Rescue in the Contemporary Era

**DOI:** 10.1093/icvts/ivaf311

**Published:** 2025-12-19

**Authors:** Michael A Catalano, Daniel Bazianos, Ashwin Nathan, Lauren Gillinov, Omar Toubat, Alexandra E Sperry, Nicholas J Goel, Nimesh D Desai, Wilson Y Szeto, Chase R Brown, Kendall M Lawrence

**Affiliations:** Division of Cardiovascular Surgery, Department of Surgery, University of Pennsylvania, Philadelphia, PA, 19104, United States; Division of Cardiovascular Surgery, Department of Surgery, University of Pennsylvania, Philadelphia, PA, 19104, United States; Division of Cardiology, Department of Medicine, University of Pennsylvania, Philadelphia, PA, 19104, United States; Division of Cardiovascular Surgery, Department of Surgery, University of Pennsylvania, Philadelphia, PA, 19104, United States; Division of Cardiovascular Surgery, Department of Surgery, University of Pennsylvania, Philadelphia, PA, 19104, United States; Division of Cardiovascular Surgery, Department of Surgery, University of Pennsylvania, Philadelphia, PA, 19104, United States; Division of Cardiovascular Surgery, Department of Surgery, University of Pennsylvania, Philadelphia, PA, 19104, United States; Division of Cardiovascular Surgery, Department of Surgery, University of Pennsylvania, Philadelphia, PA, 19104, United States; Leonard Davis Institute, University of Pennsylvania, Philadelphia, PA, 19104, United States; Division of Cardiovascular Surgery, Department of Surgery, University of Pennsylvania, Philadelphia, PA, 19104, United States; Division of Cardiovascular Surgery, Department of Surgery, University of Pennsylvania, Philadelphia, PA, 19104, United States; Leonard Davis Institute, University of Pennsylvania, Philadelphia, PA, 19104, United States; Division of Cardiovascular Surgery, Department of Surgery, University of Pennsylvania, Philadelphia, PA, 19104, United States

**Keywords:** TAVR, failure-to-rescue, surgical complications, quality improvement

## Abstract

**Objectives:**

As transcatheter aortic valve replacement (TAVR) expands to lower-risk populations, understanding contemporary patterns of complications requiring surgical intervention remains critical. This study examines the incidence, predictors, and outcomes of major TAVR complications.

**Methods:**

The National Inpatient Sample (2016-2021) was queried to identify adult patients undergoing TAVR. Major complications were defined as surgical aortic valve replacement, coronary artery bypass grafting, aortic intervention, pericardial drainage, VA-ECMO, cardiac repair, or diagnosis of aortic dissection/rupture. Multivariable logistic regression identified predictors of complications and failure-to-rescue.

**Results:**

Among 383 395 TAVRs, 4685 (1.2%) experienced major complications. Overall in-hospital mortality was 1.3%. Mortality was 26.0% in patients with major complications versus 1.0% without (*P* < .001). Stroke rates were also higher in patients with major complications (7.5% versus 1.8%, *P* < .001). Complications were associated with longer length of stay (8 vs 2 days) and higher hospital costs ($79,302 vs $45,469). Independent predictors of complications included age <65 (OR 2.27), bicuspid aortic valve (OR 1.79), thoracic aortic aneurysm (OR 1.49) and female sex (OR 1.24), while elective admission was protective (OR 0.51). Among patients with complications, VA-ECMO cannulation (OR 10.36), cardiac chamber repair (OR 3.14), and aortic dissection/rupture (OR 1.68) were strongest predictors of mortality.

**Conclusions:**

While the proportion of TAVR patients experiencing surgical emergencies has remained stable over time, the overall prevalence is increasing with the growth of TAVR, and these complications are associated with an in-hospital mortality rate of greater than 25%. Younger age, female sex, bicuspid valve, and thoracic aneurysm are associated with increased risk of major complications.

## Graphical Abstract

**Figure:**
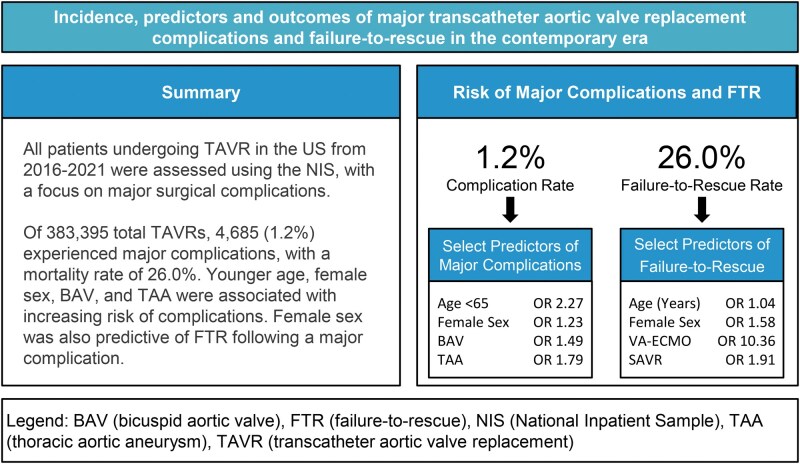

## INTRODUCTION

Transcatheter aortic valve replacement (TAVR) has revolutionized the treatment of severe aortic stenosis, and its utilization has increased rapidly in the United States over the last decade.^1–5^ Based on a growing body of evidence demonstrating excellent early and mid-term outcomes, TAVR indications have expanded to include patients of low surgical risk.^6–15^ While not yet recommended by major society guidelines, there has been a rapid increase in utilization of TAVR in low-risk patients, including those aged ≤65, as well as those with bicuspid aortic valve (BAV) anatomy.^3^^,^^5^^,^^11^^,^^12^^,^^14–16^

As TAVR continues to expand to lower-risk patient cohorts, it is vital to understand the frequency, predictors, and outcomes of major complications. Despite the overall safety profile of TAVR, prior studies have demonstrated that major complications requiring emergent open cardiac surgical intervention or mechanical circulatory support persist, and such complications carry extremely high risk of morbidity and mortality. These complications, while reported to occur in only about 1%-2% of cases, are associated with short-term mortality rates ranging from 25% to 50%^17–23^; more contemporary data on the incidence and outcomes of such complication are limited. Furthermore, the concept of failure-to-rescue (FTR), or the inability to prevent mortality following a major complication, has emerged as a key quality metric across procedural specialties.^24^

Thus, the aim of this study is to identify the incidence, predictors, and outcomes of major complications following TAVR, as well as predictors of FTR among patients experiencing such complications. By understanding these patterns in the modern era, we seek to inform patient selection, procedural planning, and quality improvement efforts as TAVR continues to expand to low-risk patient populations.

## METHODS

### Data source

The National Inpatient Sample (NIS) was queried for the years 2016 to 2021. This database contains a representative sample of all inpatient hospital admissions in the United States and is maintained by the Healthcare Cost and Utilization Project of the Agency for Healthcare Research and Quality. The NIS represents approximately 20% of all U.S. hospitalizations and is designed to be nationally representative when appropriate statistical weights are applied.

Discharge records contain patient-specific information, including age, sex, race, insurance status, diagnoses reported during the hospitalization, procedures performed during the hospitalization, length of stay, hospital charges, and mortality. Diagnoses are reported using the International Classification of Diseases, Tenth Revision, Clinical Modification (ICD-10-CM) system, and procedures are reported using the International Classification of Diseases, Tenth Revision, Procedure Coding System (ICD-10-PCS). Records also contain data on hospital size, location, and teaching status.

This study was deemed exempt from review by the University of Pennsylvania Institutional Review Board because the data is publicly available and de-identified (IRB #858879).

### Study design, population and key variables

A retrospective case-control study was conducted, comparing the exposure and outcomes of patients who underwent TAVR and experienced a pre-defined major complication, to those who underwent TAVR and did not experience a major complication.

The NIS was queried to identify all inpatient admissions of individuals aged ≥18 years who underwent TAVR during their hospital admission. TAVR was identified using ICD-10-PCS codes 02RF37H, 02RF37Z, 02RF38H, 02RF38Z, 02RF3JH, 02RF3JZ, 02RF3KH, 02RF3KZ.

Patients were then stratified according to whether they experienced a major complication during the same hospital admission. Major complications were defined based on the presence of ICD-10-CM or ICD-10-PCS codes for any of the following (**Table S1**):

- Procedure code for surgical aortic valve replacement (SAVR)
- Procedure code for coronary artery bypass grafting (CABG)
- Procedure code for replacement of ascending aorta or aortic root
- Procedure code for open drainage of pericardium or mediastinum
- Procedure code for open repair of cardiac structure
- Procedure code for open removal of intra-cardiac device
- Procedure code for veno-arterial extracorporeal membrane oxygenation (VA-ECMO)
- Diagnosis code for thoracic aortic dissection or rupture

For patients with a major complication requiring a procedure, the timing of the procedural intervention relative to the index TAVR, in days, was identified.

Patient demographics, including age, sex, and race, were reviewed. Age was assessed as a continuous variable, and as a grouped categorical variable. Comorbidities were defined using the Elixhauser comorbidity classification and methodology, derived from ICD-10-CM diagnostic codes present during inpatient admission. Other key comorbidities identified included BAV, thoracic aortic aneurysm (TAA), and aortic insufficiency (**Table S2**). Hospital characteristics, including hospital size, location, and teaching status, were also reviewed.

The primary end-point of interest was in-hospital mortality. Other end-points included in-hospital stroke as identified by ICD-10-CM codes (**Table S2**), length of stay, and measures of healthcare utilization (ie, total hospital charges and costs). Hospital charges, which represent the total amount billed at the completion of the hospital admission, were converted to costs, which represent the actual economic cost of a hospitalization, utilizing year and hospital-specific ratios.^25^

### Statistical analysis

The incidence of major TAVR complications was assessed over the study period. Descriptive patient and hospital characteristics associated with the occurrence of major complications were analyzed, including demographics, hospital size and teaching status, and comorbidity burden. Outcomes including in-hospital mortality, length of stay, and total hospital costs were assessed and compared between patients with and without major TAVR complications. Continuous variables were compared using Student’s t-test, while categorical variables were compared using chi-square tests. Statistical significance was defined as *P* < .05.

Multivariable logistic regression was utilized to assess independent predictors of the occurrence of major TAVR complications. Variables included in the model were selected based on clinical relevance and statistical significance in univariate analysis. Similarly, multivariable logistic regression was utilized to assess predictors of FTR, defined as mortality among patients who experienced a major TAVR complication. Secondarily, interaction terms between age and sex were tested in multivariable models using likelihood ratio testing to assess model improvement. When a statistically significant interaction was identified, stratified analyses were performed to further evaluate predictors of major TAVR complications within each gender subgroup.

All analyses were performed using Stata, version 18.0 (StataCorp). All *P*-values were 2-sided with a significance threshold of <0.05. A 95% confidence interval was defined as statistical significance for all analyses. Descriptive analyses incorporated survey weights as appropriate.

## RESULTS

**Figure 1.**
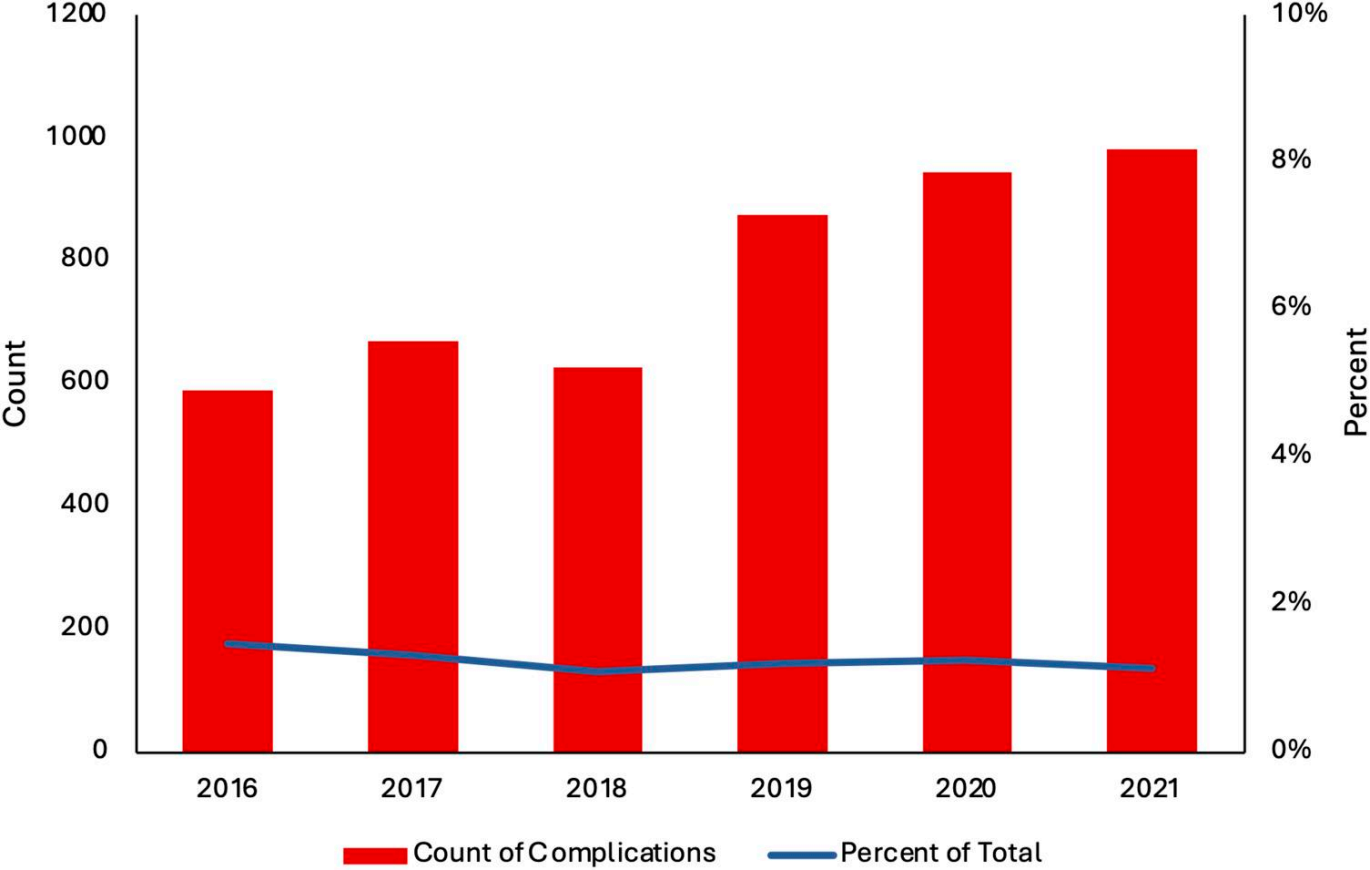
Count of Major TAVR Complications, by Year

**Figure 2.**
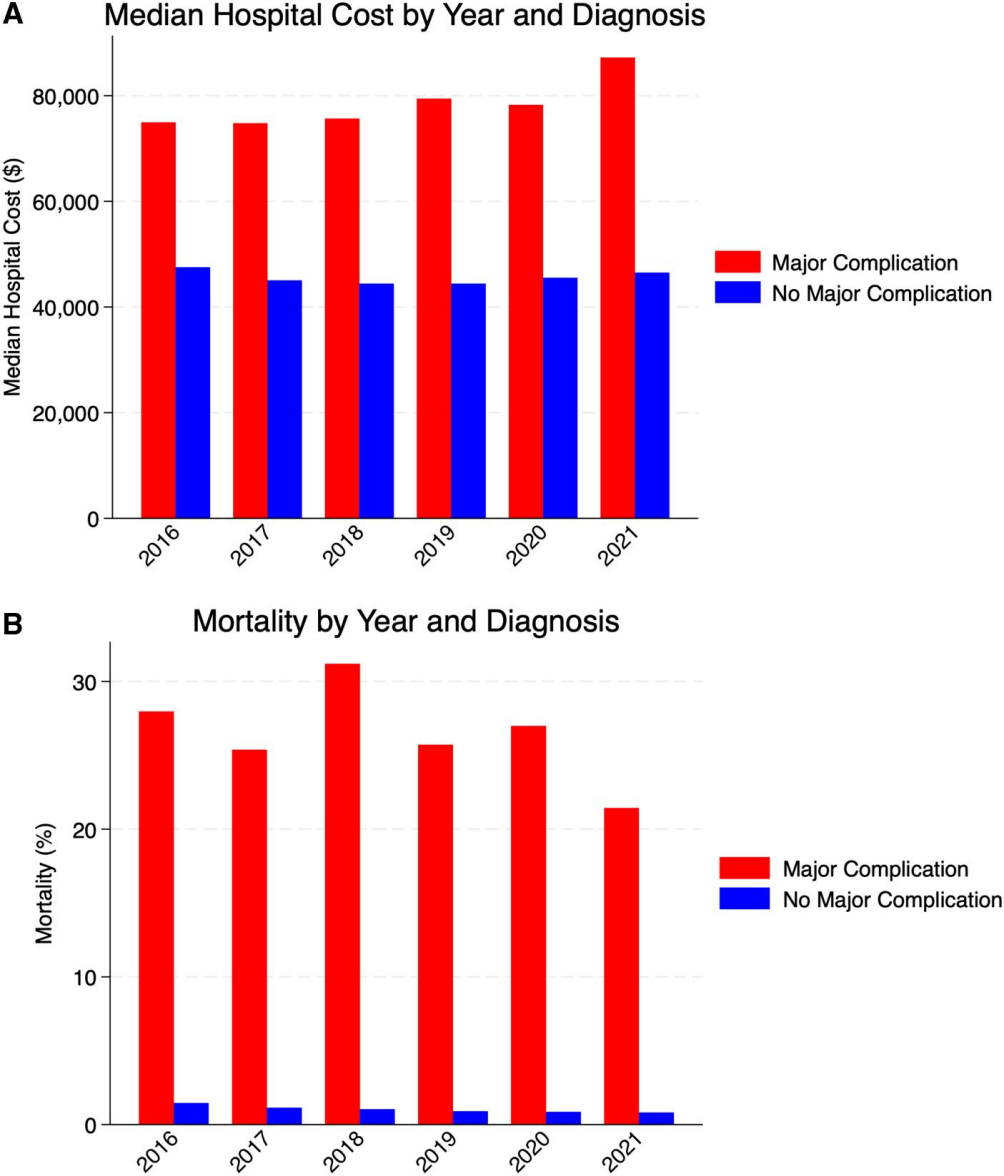
Total Hospital Costs (A) and Mortality Rate (B) Associated with Major TAVR Complications, by Year

During the study period from 2016 to 2021, a total of 383 395 weighted admissions of patients undergoing TAVR were identified. The total number of TAVRs performed annually increased substantially over the study period, from 40 000 in 2016 to 86 725 in 2021. In total, 4685 patients (1.2%) experienced a major complication as previously defined. The annual incidence of major complications remained relatively stable over the study period, ranging from 1.1% to 1.5%; however, the absolute number of major complications increased from 590 in 2016 to 980 in 2021, reflecting the expanding overall utilization of TAVR (**Figure 1**). Overall mortality rates remained relatively stable over the study period for both patients with and without major complications. Among patients with complications, mortality rates ranged from 21.0% to 31.0% across the study years, without a clear trend. Hospital costs increased over time, particularly for patients with complications (**Figure 2**).

Patients experiencing major complications differed significantly from those without complications across several key demographic and clinical characteristics (**Table 1**). The median age of patients with complications was younger (79.0 vs 80.0 years, *P* < .001), with a significantly greater concentration of young patients in the major complication cohort—patients aged <65 made up 14.3% of all patients experiencing major complications, as compared to 6.0% of patients experiencing no major complications (*P* < .001). There was also a trend towards female predominance in the complication cohort (46.9% vs 44.0%, *P* = .076). Patients with complications were less likely to be admitted electively (69.3% vs 83.2%, *P* < .001), more likely to have BAV (5.3% vs 1.9%, *P* < .001), and more frequently had concurrent TAA (5.8% vs 1.4%, *P* < .001). The presence of aortic insufficiency was also more prevalent in the complication group (19.0% vs 11.2%, *P* < .001). Interestingly, patients experiencing complications had lower rates of several traditional cardiovascular risk factors, including diabetes mellitus (27.7% vs 37.7%, *P* < .001), hypertension (84.9% vs 89.9%, *P* < .001), and chronic obstructive pulmonary disease (22.8% vs 26.2%, *P* < .001). However, they had higher rates of heart failure (75.6% vs 71.8%, *P* = .011), severe renal failure (10.7% vs 7.5%, *P* < .001), and moderate-to-severe liver disease (2.2% vs 1.0%, *P* < .001). There was also a significantly higher rate of peripheral vascular disease among patients with complications (50.8% vs 21.5%, *P* < .001). There was no statistically significant association between hospital location, teaching status, or size and occurrence of major complications.

**Table 1.** Baseline Demographics, Hospital Characteristics, and Select Comorbidities—TAVR Patients with and without Major Complications

	No Major Complication	Major Complication	*P*-value
	378 710 (98.8%)	4685 (1.2%)	
Age (IQR)	80.0 (73.0-85.0)	79.0 (70.0-85.0)	<.001
Age Group (%)			<.001
<65	22 645 (6.0%)	670 (14.3%)	
65-74	85 940 (22.7%)	995 (21.2%)	
75-84	162 905 (43.0%)	1705 (36.4%)	
≥85	107 220 (28.3%)	1315 (28.1%)	
Calendar Year (%)			.168
2016	39 410 (10.4%)	590 (12.6%)	
2017	50 490 (13.3%)	670 (14.3%)	
2018	56 530 (14.9%)	625 (13.3%)	
2019	71 745 (18.9%)	875 (18.7%)	
2020	74 790 (19.7%)	945 (20.2%)	
2021	85 745 (22.6%)	980 (20.9%)	
Sex (%)			.076
Male	212 220 (56.0%)	2490 (53.1%)	
Female	166 455 (44.0%)	2195 (46.9%)	
Race (%)			.010
White	320 965 (87.3%)	3810 (84.3%)	
Black	14 910 (4.1%)	215 (4.8%)	
Hispanic	17 570 (4.8%)	310 (6.9%)	
Asian	5080 (1.4%)	55 (1.2%)	
Native American	1075 (0.3%)	0 (0.0%)	
Other	7875 (2.1%)	130 (2.9%)	
Hospital Location/Teaching Status (%)			.189
Rural	1265 (0.9%)	10 (0.5%)	
Urban, Non-Teaching	13 785 (9.4%)	130 (6.9%)	
Urban, Teaching	131 380 (89.7%)	1745 (92.6%)	
Hospital Bed Size (%)			.180
Small	9890 (6.8%)	100 (5.3%)	
Medium	28 825 (19.7%)	320 (17.0%)	
Large	107 715 (73.6%)	1465 (77.7%)	
Sum of Elixhauser Comorbidities (IQR)	5.0 (4.0-6.0)	6.0 (4.0-7.0)	<.001
Elixhauser Comorbidities			
Cerebrovascular disease (%)	37 900 (10.0%)	755 (16.1%)	<.001
Diabetes (%)	143 005 (37.7%)	1300 (27.7%)	<.001
Heart failure (%)	271 960 (71.8%)	3540 (75.6%)	.011
Hypertension (%)	340 385 (89.9%)	3975 (84.9%)	<.001
Liver disease, moderate to severe (%)	3640 (1.0%)	105 (2.2%)	<.001
Chronic pulmonary disease (%)	99 245 (26.2%)	1070 (22.8%)	.020
Obesity (%)	79 050 (20.9%)	870 (18.6%)	.084
Peripheral vascular disease (%)	81 335 (21.5%)	2380 (50.8%)	<.001
Pulmonary circulation disease (%)	59 210 (15.6%)	805 (17.2%)	.195
Renal failure, severe (%)	28 385 (7.5%)	500 (10.7%)	<.001
Other Key Comorbidities			
Bicuspid Aortic Valve (%)	7310 (1.9%)	250 (5.3%)	<.001
Aortic Insufficiency (%)	42 435 (11.2%)	890 (19.0%)	<.001
Thoracic Aortic Aneurysm (%)	5385 (1.4%)	270 (5.8%)	<.001
Elective Hospital Admission (%)	313 880 (83.2%)	3225 (69.3%)	<.001

The most common major complication was aortic dissection or rupture, occurring in 1315 patients (28.1%, **Table 2**). Open pericardial drainage was performed in 975 patients (20.8%), VA-ECMO cannulation in 950 patients (20.3%), open aortic interventions in 780 patients (16.6%), CABG in 725 patients (15.5%), repair of cardiac chambers in 605 patients (12.9%), SAVR in 525 patients (11.2%), and open removal of intra-cardiac device in 190 patients (4.1%). The majority of procedural complications occurred early relative to TAVR. Among the 3340 patients for whom procedure dates are available for both TAVR and major complications, 2935 (87.9%) occurred on the same day as TAVR, 200 (6.0%) on post-TAVR days 1-2, 140 (3.6%) on post-TAVR days 3-7, and 65 (1.9%) on post-TAVR day >7.

**Table 2.** Complication Rates and in-Hospital Outcomes, among TAVR Patients with and without Major Complications

	No Major Complication	Major Complication	*P*-value
	378 710 (98.8%)	4685 (1.2%)	
Major TAVR Complications (%)			
SAVR	–	525 (11.2%)	–
CABG	–	725 (15.5%)	–
Open Aortic Intervention	–	780 (16.6%)	–
Open Pericardial Drainage	–	975 (20.8%)	–
VA-ECMO	–	950 (20.3%)	–
Aortic Dissection or Rupture	–	1315 (28.1%)	–
Open Repair of Cardiac Chamber	–	605 (12.9%)	–
Open Removal of Cardiac Device	–	190 (4.1%)	–
Length of Stay (IQR)	2.0 (1.0-4.0)	8.0 (4.0-15.0)	<.001
Total Hospital Costs (IQR)	45 469 (35 452-58 465)	79 302 (53 921-120 846)	<.001
In-Hospital Mortality (%)	3730 (1.0%)	1220 (26.0%)	<.001
In-Hospital Stroke (%)	6835 (1.8%)	355 (7.6%)	<.001

Patients experiencing complications had significantly higher in-hospital mortality (26.0% vs 1.0%, *P* < .001), in-hospital stroke rates (7.5% vs 1.8%, *P* < .001), length of stay (8.0 vs 2.0 days, *P* < .001), and total hospital costs ($79,302 vs $45,469, *P* < .001).

Multivariable logistic regression analysis identified several independent predictors of major TAVR complications (**Table 3**). BAV (OR 1.79, 95% CI 1.31-2.44, *P* < .001) and TAA (OR 1.49, 95% CI 1.11-2.01, *P* = .009) were notable predictors of major complications. Other factors associated with complications included female sex (OR 1.24, 95% CI 1.09-1.41, *P* = .002), age <65 (OR 2.27, 95% CI 1.86-2.77, *P* < .001), and non-elective admission status (OR 0.51 for elective admission, 95% CI 0.44-0.59, *P* < .001). A significant interaction between female sex and age was identified and confirmed using likelihood ratio testing, which supported the conduct of a secondary, gender-stratified analysis of predictors of major TAVR complications. Stratified models demonstrated that age <65 years was associated with increased odds of major TAVR complications in both females (OR 1.78, 95% CI 1.26-2.52, *P* = .001) and males (OR 2.52, 95% CI 1.96-3.23, *P* < .001), with a greater effect observed among males (**Table S4**).

**Table 3.** Multivariable Logistic Regression, Predictors of Major TAVR Complications

	OR	95% CI	*P*-value
Age <65	2.27	1.86-2.77	<.001
Female Sex	1.23	1.09-1.41	.002
White Race	0.89	0.75-1.06	.187
Elective Admission	0.51	0.44-0.59	<.001
Bicuspid Aortic Valve	1.79	1.31-2.44	<.001
Thoracic Aortic Aneurysm	1.49	1.11-2.02	.009
Diabetes with chronic complications	0.88	0.74-1.05	.161
Heart failure	1.33	1.11-1.60	.002
Hypertension, complicated	0.72	0.61-0.85	<.001
Liver disease, moderate to severe	1.90	1.22-2.98	.005
Chronic pulmonary disease	0.72	0.62-0.84	<.001
Obesity	0.90	0.76-1.07	.251
Peripheral vascular disease	3.74	3.27-4.29	<.001
Pulmonary circulation disease	0.96	0.80-1.14	.623
Renal failure, severe	1.30	1.03-1.62	.024

Descriptive analysis comparing patients with major complications who died during admission (ie, FTR) to those who survived revealed key demographic and comorbidity differences (**Table S3**). Specifically, patients who died were older (82.0 vs 78.0 years, *P* < .001) and more likely to be female (56.6% vs 43.4%, *P* < .001). There were no major differences in comorbidities, though TAA was less common among patients who died (1.6% vs 7.2%, *P* = .001). There was no statistically significant association between hospital complications and FTR. Patients who died had a significantly shorter length of stay but increased total hospital costs.

Multivariable analysis identified older age and female sex as predictors of FTR (**Table 4**). No significant interaction was observed between age and sex. There was no association between BAV or TAA diagnosis and mortality in multivariable analysis. Among specific complications, the strongest predictor of FTR was the need for VA-ECMO cannulation (OR 10.36, 95% CI 6.32-16.98, *P* < .001). Repair of cardiac chambers (OR 3.14, 95% CI 1.91-5.17 *P* < .001), SAVR (OR 1.91, 95% CI 1.01-3.63, *P* = .047), and diagnosis of aortic dissection or rupture (OR 1.68, 95% CI 1.56-4.61, *P* < .001) were also associated with increased mortality risk.

**Table 4.** Multivariable Logistic Regression, Predictors of Mortality, among Patients with Major TAVR Complications

	OR	95% CI	*P*-value
Age	1.04	1.01-1.06	.001
Female Sex	1.58	1.11-2.22	.011
Elective Admission	1.44	0.97-2.13	.068
Bicuspid Aortic Valve	0.99	0.42-2.34	.984
Thoracic Aortic Aneurysm	0.39	0.13-1.21	.103
Diabetes with chronic complications	0.82	0.53-1.28	.384
Heart failure	0.68	0.43-1.07	.095
Hypertension, complicated	0.79	0.53-1.17	.236
Liver disease, moderate to severe	3.04	1.12-8.28	.029
Chronic pulmonary disease	0.85	0.57-1.28	.441
Obesity	0.82	0.51-1.31	.403
Peripheral vascular disease	1.01	0.64-1.59	.963
Pulmonary circulation disease	1.34	0.87-2.05	.183
Renal failure, severe	1.38	0.81-2.34	.233
SAVR	1.91	1.01-3.63	.047
CABG	1.16	0.64-2.09	.623
Open Aortic Intervention	1.64	0.96-2.81	.071
Open Pericardial Drainage	1.52	0.96-2.41	.075
VA-ECMO	10.36	6.32-16.98	<.001
Aortic Dissection or Rupture	1.68	1.56-4.61	<.001
Open Repair of Cardiac Chamber	3.14	1.91-5.17	<.001
Open Removal of Cardiac Device	0.80	0.30-2.12	.647

## DISCUSSION

In this contemporary national analysis of TAVR practice and outcomes major surgical complications remained stable at about 1.2%, while mortality (26.0%) and stroke rates (7.5%) remained high among affected patients. Though complication rates were unchanged over time, the absolute number of patients affected increased nearly twofold due to the rapid expansion of TAVR utilization. Factors typically associated with lower risk patients—younger age, BAV, and lesser comorbidity burden—are associated with increased risk of complications. Notably, female patients faced both higher complication rates and increased failure-to-rescue (FTR).

Over the last decade, TAVR utilization has increased rapidly in the United States^1–4^ based on the non-inferiority of TAVR versus SAVR demonstrated by key randomized-controlled trials,^6–10^^,^^13^ with growth driven by inclusion of intermediate- and low-risk patients.^3^^,^^5^ However, it is important to acknowledge that further changes in practice are outpacing relevant changes in guidelines. For example, while United States and European guidelines continue to recommend SAVR over TAVR in patients aged <65 and <75, respectively,^26^^,^^27^ recent data from the California, New York, and New Jersey state databases revealed that 54.7% of patients aged <65 undergoing aortic valve replacement underwent TAVR in 2021.^5^ Similarly, while the 2020 ACC/AHA guidelines recommend SAVR in low-risk patients with BAV,^27^ real-world evidence continues to demonstrate increasing use of TAVR in patients with BAV.^5^^,^^14^^,^^16^^,^^28^ This is particularly notable given the exceedingly low risk of SAVR mortality in young BAV patients.^29^

FTR, defined as a mortality that occurs after a major complication, has emerged as a key quality metric across surgical specialties, as it is suggestive of the surgical team’s ability to respond to complex post-operative scenarios.^24^^,^^30–33^ While FTR has previously been defined in the cardiac surgical literature as a mortality that occurs after a broad range of complications, including infection, stroke, and renal failure, the aim of this study is to assess the procedural risks that are unique to TAVR—specifically, the risk of injury to major cardiac or vascular structures that may require emergent surgical conversion or cardiopulmonary support. In contrast to open cardiac surgical procedures, in which key structures are exposed and accessible, such complications during TAVR require rapid intervention, often with the patient in unstable condition. For that reason, we define major complications as any event that would typically require surgical intervention—including not only patients who underwent sternotomy, but also patients with aortic dissection or rupture who may have been deemed inoperable, as well as those requiring cardiopulmonary support with VA-ECMO. Using this definition, we have identified a stable rate of major complications of 1.2%, with an associated in-hospital mortality rate of 26.0% and a stroke rate of 7.5%.

These findings are largely consistent with the literature, with major differences related to varied definitions of complications and FTR. For example, Aarts and colleagues, utilizing a multicentre registry of 24 010 TAVR patients from 2007 to 2022, identified a 0.5% rate of surgical bailout, which decreased over the study period.^17^ Interestingly, we identified a stable rate of major complications over time; this suggests that, despite the introduction of lower risk patients and increasing operator comfort, there was likely an increase in anatomic and procedural complexity that increased risk. Similar to the Aarts study, Pineda and colleagues identified a surgical bailout rate of 1.2% and a FTR rate of 50.0%.^19^ In contrast, Bishawi and colleagues assessed FTR in TAVR from 2011 to 2016 with a broader definition of complications, including pacemaker requirement, renal failure, stroke, major bleeding, and vascular injury, in addition to surgical conversion. In doing so, they identified a complication rate of 24.3% with a FTR rate of 9.6%, reflecting the higher prevalence of these less morbid post-operative complications.^23^ Similarly, Ando and colleagues, utilized a broader definition of complications in their NIS review of FTR in TAVR from 2011 to 2015, including major bleeding, vascular injury, pacemaker requirement, renal failure, and stroke, among others—as a result, they reported a complication rate of over 20% and a FTR rate of 12.3%.^18^

In multivariable analysis, we have identified several key factors typically associated with low-risk TAVR patients as predictive of major complications, including younger age, BAV, and TAA. These associations have been suggested by prior studies, including the NOTION trial, which identified an increased risk of the composite end-point of death, stroke, and rehospitalization in BAV patients undergoing TAVR.^14^ BAVs tend to be more heavily calcified, which may increase the risk of coronary obstruction or annular rupture upon TAVR deployment; moreover, the dimensions of the aortic annulus for BAVs tends to be larger, which may increase risk of valve migration.^34^^,^^35^ TAA has also been associated with increased risk of TAVR complications, including aortic dissection, major bleeding, and tamponade, which may be explained by the presence of more fragile aortic tissue and abnormal root geometry that challenges valve deployment.^36^ The counterintuitive finding that younger patients and those with fewer traditional cardiovascular comorbidities experienced higher complication rates may reflect a selection bias whereby these patients have more complex anatomical features. Second, young patients are more likely to present with BAV, which carries its own previously described risk. This finding highlights the importance of comprehensive patient assessment and highlights that traditional surgical risk assessment that emphasizes age may not fully capture procedural risk in the TAVR population.

Among patients who experienced complications, older age and female sex were associated with increased risk of in-hospital mortality. The association between female sex and FTR is an important insight into sex-related healthcare disparities, consistent with the broader surgical literature.^24^ Within the TAVR literature, specifically, female sex has been associated with both risk of complications and risk of mortality.^17^^,^^19^^,^^37^^,^^38^ While the increased risk of complications among female patients is likely related to anatomic features such as vascular access dimensions and aortic root geometry, the higher rate of FTR is indicative of disparities in the ability to salvage patients from complications, which is important to investigate further. In addition to sex and age, the need for cardiopulmonary support with VA-ECMO, as well as complications associated with major cardiovascular injury (ie, aortic dissection or rupture, and open repair of cardiac chamber) were associated with FTR. BAV and TAA diagnosis were not associated with risk of FTR.

Notably, we have not identified any association between hospital location, size, and teaching status and FTR. This finding is contrary to recent data highlighting the impact of center size and experience on ability to rescue patients from major complications.^39^^,^^40^ The reason for this is likely twofold. First, the structure of the NIS dataset does not allow a quanitifcation of individual center procedural volume, which has been demonstrated to be more associated with FTR than general hospital characteristics.^39^^,^^40^ Second, our definition of major complications, focused primarily on patients who undergo major surgical intervention, likely already selects for hospitals that have greater procedural capabilities.

There are several important limitations that should be acknowledged. First, while the NIS database provides a unique insight into real-world practice patterns and outcomes that strengthens this study, the administrative nature of the database limits the granularity of clinical detail available. For example, the ICD-10-CM system lacks relevant details on valvular anatomic considerations including BAV anatomy, the distinction between native and valve-in-valve TAVR, and the size and anatomy of TAA; additionally, ICD-10-PCS coding does not allow for identification of TAVR valve choice or access site. Similarly, details on the reason for operative intervention, (e.g., coronary occlusion, annular rupture, ventricular perforation) are not available, nor are specific valve-related complications that would allow the application of Valve Academic Research Consortium (VARC) criteria and definitions for device outcomes. Additionally, there exists the possibility of misclassification bias related to coding inaccuracy during the data entry process—a known limitation of the NIS database. Second, assessment of outcomes is limited to single inpatient hospital admissions, with an inability to track patients before or after their index hospitalization. Third, while hospital size and teaching status were not independently associated with complication rate or FTR, the structure of the NIS does not allow for quanitification of individual hospital procedural volume, which may be associated with the ability to rescue patients from major complications. Fourth, the observational nature of the study limits causal inferences, and unmeasured confounding may influence the observed associations despite controlling for numerous comorbidities in multivariable analysis. Fifth, the large sample size of the dataset may lead to analyses attributing statistical significance to small differences between groups, which may not indicate clinical significance. Finally, our analysis only includes data through 2021 and does not reflect advances in TAVR technology and techniques that may have occurred since then.

Despite these limitations, our study represents the largest and most comprehensive real-world assessment of TAVR complications and FTR outcomes in the United States in the contemporary era. In doing so, we have found that major TAVR complications continue to occur at a rate of approximately 1.2% and are associated with a mortality rate of over 25%. BAV, TAA, younger age, and female sex are associated with increased risk of complications, and older patients, female patients, and those requiring extensive surgical intervention are at greatest risk of FTR. These findings represent important considerations as TAVR continues to expand to lower risk populations and as the role of each member of the heart team is assessed.

## Supplementary Material

ivaf311_Supplementary_Data

## Data Availability

The data utilized for this study was provided by the Healthcare Cost and Utilization Project (HCUP) of the Agency for Healthcare Research and Quality, following the protocols of an institutional Data Use Agreement. Data is available from HCUP upon request.
