# Supplementary material for: Incidence, Predictors, and Outcomes of Major Transcatheter Aortic Valve Replacement (TAVR) Complications and Failure-to-Rescue in the Contemporary Era
Source: Interdiscip Cardiovasc Thorac Surg. 2025 Dec 19;41(1):ivaf311. doi: 10.1093/icvts/ivaf311 (PMC12828281; doi:10.1093/icvts/ivaf311)
Supplement: ivaf311_Supplementary_Data [file ivaf311_supplementary_data.docx]

**SUPPLEMENTARY TABLES**

Supplementary Table 1. ICD-10-PCS and ICD-10-CM codes utilized to identify major TAVR complications, including surgical aortic valve replacement, coronary artery bypass grafting, ascending aortic or root replacement, open drainage of pericardium or mediastinum, open repair of cardiac structures, open removal of intra-cardiac device, ECMO, or thoracic aortic dissection or rupture.

| **Complication** | **ICD-10-PCS or ICD-10-CM Codes** |
| --- | --- |
| Surgical aortic valve replacement (SAVR) | 02RF07Z, 02RF08Z, 02RF0KZ, 02RF0JZ |
| Coronary artery bypass grafting (CABG) | 02100*, 02110*, 02120*, 02130* |
| Replacement of ascending aorta or aortic root | 024F07J, 024F08J, 024F0JJ, 024F0KJ, 02RX07Z, 02RX08Z, 02RX0JZ, 02RX0KZ, 02RX47Z, 02RX48Z, 02RX4JZ, 02RX4KZ, 02UX07Z, 02UX08Z, 02UX0JZ, 02UX0KZ, 02UX37Z, 02UX38Z, 02UX3JZ, 02UX3KZ, 02UX47Z, 02UX48Z, 02UX4JZ, 02UX4KZ, 02VX0CZ, 02VX0DZ, 02VX0EZ, 02VX0FZ, 02VX0ZZ, 02VX3CZ, 02VX3DZ, 02VX3EZ, 02VX3FZ, 02VX3ZZ, 02VX4CZ, 02VX4DZ, 02VX4EZ, 02VX4FZ, 02VX4ZZ, 025X0ZZ, 025X3ZZ, 025X4ZZ, 027X04Z, 027X0DZ, 027X0ZZ, 027X34Z, 027X3DZ, 027X3ZZ, 027X44Z, 027X4DZ, 027X4ZZ, 02BX0ZX, 02BX0ZZ, 02BX3ZX, 02BX3ZZ, 02BX4ZX, 02BX4ZZ, 02CX0ZZ, 02CX3ZZ, 02CX4ZZ, 025X0ZZ, 025X3ZZ, 025X4ZZ, 02NX0ZZ, 02NX3ZZ, 02NX4ZZ, 02QX0ZZ, 02QX3ZZ, 02QX4ZZ, 02SX0ZZ |
| Open drainage of pericardium or mediastinum | 0W9C0*, 0W9D0* |
| Open repair of cardiac chamber or structure | 02Q60ZZ, 02Q70ZZ, 02QA0ZZ, 02QB0ZZ, 02QC0ZZ, 02QK0ZZ, 02QL0ZZ |
| Open removal of intra-cardiac device | 02PA0YZ, 02PA08Z, 02PA0DZ, 02PA0JZ, 02PA0KZ |
| Extra-corporeal membrane oxygenation | 5A15223, 5A1522F, 5A1522G, 5A15A2F, 5A15A2G |
| Thoracic aortic dissection or rupture | I7100, I7101, I7102, I7103, I711, I715 |

Supplementary Table 2. ICD-10-CM codes utilized to identify major comorbidities and diagnosis of in-hospital stroke.

| **Comorbidity** | **ICD-10-CM Codes** |
| --- | --- |
| Bicuspid aortic valve | Q230, Q231 |
| Aortic insufficiency | Q231, I351, I352 |
| Stroke | I60*, I61*, I62*, I63* |

Supplementary Table 3. Characteristics of patients experiencing major TAVR complications, stratified by in-hospital mortality

|  | Survived | Died | P-value |
| --- | --- | --- | --- |
|  | 3,465 (74.0%) | 1,220 (26.0%) |  |
| Age | 78.0 (69.0-85.0) | 82.0 (73.0-87.0) | <0.001 |
| Age Group (%) |  |  | 0.003 |
| <65 | 560 (16.2%) | 110 (9.0%) |  |
| 65-74 | 760 (21.9%) | 235 (19.3%) |  |
| 75-84 | 1,265 (36.5% | 440 (36.1%) |  |
| ≥85 | 880 (25.4%) | 435 (35.7%) |  |
| Sex |  |  |  |
| Male | 1,960 (56.6%) | 530 (43.4%) | <0.001 |
| Female | 1,505 (43.4%) | 690 (56.6%) |  |
| Race |  |  | 0.642 |
| White | 2,810 (84.0%) | 1,000 (85.1%) |  |
| Black | 180 (5.4%) | 35 (3.0%) |  |
| Hispanic | 225 (6.7%) | 85 (7.2%) |  |
| Asian | 40 (1.2%) | 15 (1.3%) |  |
| Other | 90 (2.7%) | 40 (3.4%) |  |
| Primary Payer |  |  | 0.172 |
| Medicare | 2,760 (79.7%) | 1,040 (85.2%) |  |
| Medicaid | 130 (3.8%) | 35 (2.9%) |  |
| Private | 450 (13.0%) | 120 (9.8%) |  |
| Self | 25 (0.7%) | 15 (1.2%) |  |
| Unknown | 100 (2.9%) | 10 (0.8%) |  |
| Household Income Quartile |  |  | 0.903 |
| First | 775 (22.8%) | 250 (20.7%) |  |
| Second | 845 (24.9%) | 300 (24.9%) |  |
| Third | 950 (27.9%) | 360 (29.9%) |  |
| Fourth | 830 (24.4%) | 295 (24.5%) |  |
| Hospital Location / Teaching Status |  |  | 0.667 |
| Rural | 10 (0.7%) | 0 (0.0%) |  |
| Urban, Non-Teaching | 95 (7.0%) | 35 (6.6%) |  |
| Urban, Teaching | 1,250 (92.3%) | 495 (93.4%) |  |
| Hospital Bed Size |  |  | 0.055 |
| Small | 85 (6.3%) | 15 (2.8%) |  |
| Medium | 195 (14.4%) | 125 (23.6%) |  |
| Large | 1,075 (79.3%) | 390 (73.6%) |  |
| Elective Hospital Admission | 2,330 (67.7%) | 895 (73.7%) | 0.085 |
| Bicuspid Aortic Valve | 205 (5.9%) | 45 (3.7%) | 0.184 |
| Aortic Insufficiency | 700 (20.2%) | 190 (15.6%) | 0.114 |
| Thoracic Aortic Aneurysm | 250 (7.2%) | 20 (1.6%) | 0.001 |
| Sum of Elixhauser Comorbidities | 6.0 (4.0-7.0) | 5.0 (4.0-7.0) | 0.202 |
| Cerebrovascular disease | 545 (15.7%) | 210 (17.2%) | 0.588 |
| Diabetes with chronic complications | 705 (20.3%) | 220 (18.0%) | 0.435 |
| Heart failure | 2,605 (75.2%) | 935 (76.6%) | 0.649 |
| Hypertension, complicated | 2,280 (65.8%) | 750 (61.5%) | 0.225 |
| Liver disease, moderate to severe | 65 (1.9%) | 40 (3.3%) | 0.204 |
| Chronic pulmonary disease | 830 (24.0%) | 240 (19.7%) | 0.171 |
| Obesity | 690 (19.9%) | 180 (14.8%) | 0.075 |
| Peripheral vascular disease | 1,780 (51.4%) | 600 (49.2%) | 0.556 |
| Pulmonary circulation disease | 565 (16.3%) | 240 (19.7%) | 0.231 |
| Renal failure, severe | 365 (10.5%) | 135 (11.1%) | 0.817 |
| Major TAVR Complications |  |  |  |
| SAVR | 375 (10.8%) | 150 (12.3%) | 0.531 |
| CABG | 620 (17.9%) | 105 (8.6%) | <0.001 |
| Open Aortic Intervention | 610 (17.6%) | 170 (13.9%) | 0.186 |
| Open Pericardial Drainage | 715 (20.6%) | 260 (21.3%) | 0.823 |
| VA-ECMO | 490 (14.1%) | 460 (37.7%) | <0.001 |
| Aortic Dissection or Rupture | 940 (27.1%) | 375 (30.7%) | 0.281 |
| Open Repair of Cardiac Chamber | 355 (10.2%) | 250 (20.5%) | <0.001 |
| Open Removal of Cardiac Device | 145 (4.2%) | 45 (3.7%) | 0.736 |
| Length of Stay | 9.0 5.0-16.0 | 3.0 0.0-10.0 | <0.001 |
| Total Hospital Costs | 77,046 (53,492-115,421) | 86,544 (58,532-140,631) | 0.003 |

Supplementary Table 4. Multivariable logistic regression, predictors of major TAVR complications, stratified by sex based on significance of interaction between age group and sex.

|  | **OR** | **95% CI** | **P-value** |
| --- | --- | --- | --- |
| **Male Sex** | | | |
| Age <65 | 2.52 | 1.96-3.23 | <0.001 |
| White Race | 0.70 | 0.56-0.88 | 0.002 |
| Elective Admission | 0.38 | 0.32-0.46 | <0.001 |
| Bicuspid Aortic Valve | 2.07 | 1.43-2.99 | <0.001 |
| Thoracic Aortic Aneurysm | 1.51 | 1.06-2.16 | 0.022 |
| Diabetes with chronic complications | 0.94 | 0.75-1.18 | 0.579 |
| Heart failure | 1.06 | 0.82-1.36 | 0.641 |
| Hypertension, complicated | 0.75 | 0.60-0.95 | 0.016 |
| Liver disease, moderate to severe | 2.47 | 1.50-4.06 | <0.001 |
| Chronic pulmonary disease | 0.66 | 0.53-0.83 | <0.001 |
| Obesity | 0.85 | 0.66-1.10 | 0.222 |
| Peripheral vascular disease | 4.10 | 3.40-4.95 | <0.001 |
| Pulmonary circulation disease | 0.95 | 0.74-1.22 | 0.682 |
| Renal failure, severe | 1.17 | 0.87-1.58 | 0.303 |
| **Female Sex** | | | |
| Age <65 | 1.78 | 1.26-2.52 | 0.001 |
| White Race | 1.20 | 0.92-1.56 | 0.187 |
| Elective Admission | 0.74 | 0.59-0.93 | 0.011 |
| Bicuspid Aortic Valve | 1.29 | 0.71-2.35 | 0.406 |
| Thoracic Aortic Aneurysm | 1.36 | 0.76-2.44 | 0.302 |
| Diabetes with chronic complications | 0.82 | 0.63-1.07 | 0.146 |
| Heart failure | 1.72 | 1.30-2.27 | <0.001 |
| Hypertension, complicated | 0.68 | 0.54-0.87 | 0.002 |
| Liver disease, moderate to severe | 0.79 | 0.25-2.50 | 0.690 |
| Chronic pulmonary disease | 0.80 | 0.64-0.99 | 0.046 |
| Obesity | 0.97 | 0.77-1.23 | 0.811 |
| Peripheral vascular disease | 3.38 | 2.78-4.11 | <0.001 |
| Pulmonary circulation disease | 0.96 | 0.75-1.23 | 0.729 |
| Renal failure, severe | 1.49 | 1.06-2.11 | 0.023 |
